# Heritability and genome-wide association analysis of renal sinus fat accumulation in the Framingham Heart Study

**DOI:** 10.1186/1471-2350-12-148

**Published:** 2011-11-01

**Authors:** Meredith C Foster, Qiong Yang, Shih-Jen Hwang, Udo Hoffmann, Caroline S Fox

**Affiliations:** 1 National Heart, Lung, and Blood Institute's Framingham Heart Study, 73 Mt. Wayte Avenue, Suite 2, Framingham, Massachusetts, 01702, USA; 2 Center for Population Studies, National Heart, Lung, and Blood Institute, National Institutes of Health, Bethesda, Maryland, USA; 3 Department of Epidemiology, Harvard School of Public Health, Boston, 677 Huntington Avenue, Boston, Massachusetts, 02115, USA; 4 Department of Biostatistics, Boston University School of Public Health, 801 Massachusetts Avenue, 3rd Floor, Boston, MA 02118, USA; 5 Department of Radiology, Massachusetts General Hospital, 55 Fruit Street, Boston, Massachusetts, 02114, USA; 6 Division of Endocrinology and Metabolism, Brigham and Women's Hospital, Harvard Medical School, Boston, Massachusetts, USA

## Abstract

**Background:**

Ectopic fat accumulation in the renal sinus is associated with chronic kidney disease and hypertension. The genetic contributions to renal sinus fat accumulation in humans have not been well characterized.

**Methods:**

The present analysis consists of participants from the Framingham Offspring and Third Generation who underwent computed tomography; renal sinus fat and visceral adipose tissue (VAT) were quantified. Renal sinus fat was natural log transformed and sex- and cohort-specific residuals were created, adjusted for (1) age, (2) age and body mass index (BMI), and (3) age and VAT. Residuals were pooled and used to calculate heritability using variance-components analysis in SOLAR. A genome-wide association study (GWAS) for renal sinus fat was performed using an additive model with approximately 2.5 million imputed single nucleotide polymorphisms (SNPs). Finally, we identified the associations of renal sinus fat in our GWAS results with validated SNPs for renal function (n = 16), BMI (n = 32), and waist-to-hip ratio (WHR, n = 14), and applied a multi-SNP genetic risk score method to determine if the SNPs for each renal and obesity trait were in aggregate associated with renal sinus fat.

**Results:**

The heritability of renal sinus fat was 39% (p < 0.0001); results were not materially different after adjustment for BMI (39%) or VAT (40%). No SNPs reached genome-wide significance in our GWAS. In our candidate gene analysis, we observed nominal, direction consistent associations with renal sinus fat for one SNP associated with renal function (p = 0.01), two associated with BMI (p < 0.03), and two associated with WHR (p < 0.03); however, none remained significant after accounting for multiple testing. Finally, we observed that in aggregate, the 32 SNPs associated with BMI were nominally associated with renal sinus fat (multi-SNP genetic risk score p = 0.03).

**Conclusions:**

Renal sinus fat is a heritable trait, even after accounting for generalized and abdominal adiposity. This provides support for further research into the genetic determinants of renal sinus fat. While our study was underpowered to detect genome-wide significant loci, our candidate gene BMI risk score results suggest that variability in renal sinus fat may be associated with SNPs previously known to be associated with generalized adiposity.

## Background

In the midst of a global obesity epidemic, current evidence suggests that ectopic fat deposition, or fat accumulation within and around non-adipose tissue and organs [1], plays an important role in the development of obesity-related disease and local organ dysfunction. In the Framingham Heart Study, we have observed that several ectopic fat depots are independently associated with cardiometabolic risk above and beyond measures of general obesity, including abdominal visceral adipose tissue [2], pericardial fat [3], fatty liver [4], upper body subcutaneous fat [5], and peri-aortic fat [6].

Obesity is also implicated in the development of chronic kidney disease (CKD) [7-13], a common condition that affects over 13% of adults in the United States [14]. In animal models, fat accumulation within the renal sinus may play a mediating role between obesity and renal function [1]. We have recently characterized this novel ectopic fat depot in humans, and observed that high renal sinus fat accumulation, or "fatty kidney," is associated with chronic kidney disease even after accounting for other adiposity measures [15].

While the genetic components associated with ectopic fat depots such as renal sinus fat have not been well studied, a growing body of evidence supports the hypothesis that an independent genetic contribution to ectopic fat deposition exists beyond those implicated in general obesity. Increased ectopic fat accumulation is a common clinical characteristic that accompanies the loss of traditional adipose tissue stores in inherited lipodystrophies, and several genetic loci are implicated in the pathogenesis of these disorders [16-18]. Measures of regional and ectopic fat accumulation, including waist circumference [19], waist-to-hip ratio (WHR) [20,21], and ectopic fat in skeletal muscle [22], are heritable even after adjusting for body mass index (BMI). Finally, several genetic loci have also been identified in genome-wide association studies (GWAS) that are associated with anthropometric measures of regional, but not overall, obesity [23,24].

Thus, we sought to characterize the genetic contribution to renal sinus fat by assessing heritability and performing a GWAS in the Framingham Heart Study. As an ectopic fat depot that is associated with chronic kidney disease, we also performed a candidate gene analysis to assess the association of renal sinus fat with genetic loci recently identified in association with renal function [25] and adiposity measures [24,26].

## Methods

### Study sample

The study sample was comprised of participants from the Framingham Heart Study Offspring and Third Generation cohorts who underwent multi-detector computed tomography (MDCT) between 2002 and 2005. The MDCT protocol and eligibility criteria have been described previously [2]. Of the 3529 participants who underwent MDCT, 2978 attended either the 7^th ^Offspring (1998-2001, N = 1259) or the 1^st ^Third Generation (2002-2005, N = 1719) examination and had an MDCT scan interpretable for the renal sinus fat measurement. Participants provided written informed consent, and this study was approved by the institutional review boards at the Boston University Medical Center and Massachusetts General Hospital. In the heritability analysis, participants without additional family members in the sample were excluded in order to evaluate family correlations, resulting in a sample size of 2946 participants (1489 women and 1457 men). For the GWAS and candidate gene analysis, complete genotype data were available for 2809 participants (1422 women, 1387 men).

### Phenotype definition

Renal sinus fat (cm^2^) was quantified in the right kidney based on a single slice abdominal MDCT scan. In brief, a representative slice of the renal sinus in the right kidney was identified by the reader after identifying candidate slices based on visual inspection and applying a selection rule. This measurement protocol has good intra- and inter-reader reproducibility, with intra-class correlation coefficients of 0.93 and 0.86, respectively [27]. Different tissues appear in MDCT scans with unique pixel densities in Hounsfield Units (HU), and adipose tissue was identified based on a pixel density ranging from -195 to -45 HU, centered on -120 HU.

### Additional covariate assessment

Abdominal visceral adipose tissue volume (VAT, cm^3^) was assessed by abdominal MDCT, as previously described [2]. Age and BMI (kg/m^2^) were determined during the 7^th ^Offspring or 1^st ^Third Generation examination cycles. BMI was determined using weight and height measurements obtained by trained clinic personnel. Obesity was defined as a BMI of at least 30 kg/m^2^. Hypertension was defined as a systolic blood pressure ≥140 mmHg, a diastolic blood pressure ≥90 mmHg as measured based on the mean of two readings by a clinic physician, or current use of hypertension medication. Chronic kidney disease was defined as the presence of an estimated glomerular filtration rate < 60 mL/min/1.73 m^2 ^based on the abbreviated Modification of Diet in Renal Disease Study Equation [28]. Diabetes was defined as the presence of a fasting plasma glucose ≥126 mg/dL or current use of diabetes medication.

### Genotyping and Imputation

In the Framingham SNP Health Association Resource (Genetics and Genomics Program, SHARe: SNP Health Association Resource: http://public.nhlbi.nih.gov/GeneticsGenomics/home/share.aspx), approximately 550,000 single nucleotide polymorphisms (SNPs) were genotyped with Affymetrix 500 K and 50 K gene-centric genotyping arrays (Affymetrix, Inc.; Santa Clara, CA). The genotyped SNPs and a reference panel based on the Phase II CEU HapMap were then used to create an imputed set of approximately 2.5 million SNPs with MACH version 1.0.15 imputation software.

### Statistical Analyses

Renal sinus fat (cm^2^) was analyzed as a continuous trait and, due to its skewed distribution, was natural log transformed before residual creation. The lower limit of detection was 0.0048 cm^2^; renal sinus fat measurements below 0.0048 cm^2 ^were set to 0.004 cm^2 ^before applying the log transformation. Sex- and cohort-specific residuals for renal sinus fat were created, adjusted for (1) age, (2) age and BMI, and (3) age and VAT.

Heritability of renal sinus fat in the total study sample and separately among women and men was estimated in SOLAR (Texas Biomedical Research Institute; San Antonio, TX) using the residuals described above, with additive genetic variation calculated using variance-components analysis. This measurement estimates the proportion of the total variability in renal sinus fat attributable to genotypic variability.

We performed our GWAS for continuous renal sinus fat (sex- and cohort-specific residuals, adjusted for age and BMI) using a linear mixed effects model to identify additive genetic associations with our set of approximately 2.5 million imputed SNPs from Framingham SHARe. We performed our linear mixed models using R version 2.9.2 and the lmekin function in the GWAF package and accounted for the correlation between related individuals by including family specific random effects with twice the kinship coefficient matrix as their correlations, thus accounting for arbitrary pedigree structures. A p-value of 5 × 10^-8 ^was used as the threshold for genome-wide significance. In our GWAS results, top independent SNPs were defined as SNPs with an r^2 ^< 0.2 with other SNPs in a given genomic region (i.e. when several SNPs r^2 ^> 0.20 were identified among our top SNPs, the SNP with the lowest p-value was selected to represent this genetic locus). We performed this analysis in the total sample as well as separately among women and men. Given sex-based differences observed for other regional fat depots [24], we also performed the GWAS for renal sinus fat adjusted for age and BMI among men and women separately. We identified the top five SNPs in women and men separately and determined the beta coefficient and p-value for each SNP in the opposite sex. All final results were filtered on low imputation score (< 0.3) or minor allele frequency less than 5%. We estimate that we have 80-90% power to detect a 1.3% to 1.5% variation in our renal sinus fat phenotype explained by a single SNP. In order to detect variants that explain a similar variance in renal sinus fat as for the strongest reported adiposity variant (rs9939609 in the *FTO *gene, explaining about 1% of the variance in BMI [29]) with 80-90% power, we estimate that we would require a sample size of 3750 to 4400 individuals.

The candidate gene analysis was performed in the overall sample based on our GWAS using age-adjusted residuals. We investigated the association of renal sinus fat with renal function and obesity-related SNPs previously validated in large GWAS meta-analyses, including SNPs known to be associated with renal function (n = 16)[25], BMI (n = 32) [26], and WHR (n = 14) [24]. We determined one-sided p-values to test for direction-consistent associations for SNPs in this analysis and then corrected for multiple testing by applying a false-discovery rate (FDR) analysis and determining the q-values as proposed by Storey and Tibshirani [30]. The q-value can be interpreted as the proportion of significant results that are false positives, and we considered a q-value≤0.05 as significant (i.e. at this threshold, we expect that 5% of the observed significant associations are false positives). The FDR analyses were performed using the 'qvalue' package [30] in R version 2.9.2.

Finally, we used a multi-SNP genetic risk score approach to determine if each set of SNPs associated with (1) renal function (n = 16 SNPs), (2) BMI (n = 32 SNPs), or (3) WHR (n = 14 SNPs) were associated with renal sinus fat. In brief, this method involves the creation of a risk score in our study sample based on linear regression, with continuous renal sinus fat modeled separately as a function of the three sets of SNPs above. With this regression model, by assuming that the set of SNPs used as explanatory variables are uncorrelated and that a relatively small proportion of the variance in the phenotype explained by these SNPs, we can use (1) effect estimates for each SNP from the prior GWAS analyses and (2) the effect estimates from out renal sinus fat GWAS and (3) the standard errors for the effect estimates for each SNP in our renal sinus fat GWAS, to maximize the likelihood function and create a summary test statistic across the candidate SNPs to determine whether these SNPs in aggregate are associated with renal sinus fat in our study sample. This test statistic is distributed χ2 with one degree of freedom, and we determined whether this test statistic was significant by comparing it to a critical value of $\chi_{\alpha =0.05,1df}^{2}=3.84$. This method is a cross-phenotype approach that allows us to identify potential enrichment for the association of candidate genes with renal sinus fat in our dataset and gives us an opportunity to improve the power of our individual SNP approach.

## Results

### Study sample characteristics

Overall, the study sample consisted of 2978 participants (50.8% women) with an average age of 51 years, average BMI of 27.6 kg/m^2^, and a median renal sinus fat measurement of 0.31 cm^2^. Sex-specific characteristics of the study sample are presented Table 1.

**Table 1:** Study sample characteristics by sex.

	Women	Men
	n = 1512	n = 1466
Age (years)	52 ± 10	51 ± 11
Renal sinus fat (cm^2^)*	0.20 [0.05, 0.45]	0.48 [0.21, 0.95]
Body Mass Index (kg/m^2^)	27.0 ± 5.9	28.3 ± 4.6
Abdominal visceral adipose tissue (cm^3^)	1361 ± 836	2257 ± 1047
Obesity (%)	381 (25.2%)	409 (27.9%)
Hypertension (%)	405 (26.8%)	475 (32.4%)
Diabetes (%)	75 (5.0%)	100 (6.8%)
Chronic Kidney Disease (%)	60 (4.0%)	36 (2.5%)
Current Smoking (%)	188 (12.4%)	206 (14.1%)

Continuous characteristics are presented as mean ± standard deviation and dichotomous characteristics are presented as N (%), unless otherwise noted.

* Renal sinus fat presented as median [25^th ^percentile, 75^th ^percentile]

### Heritability of renal sinus fat

The overall estimated heritability of renal sinus fat adjusted for age was 39% (p < 0.0001, Table 2) and was essentially unchanged after additional adjustment for BMI (39%) or VAT (40%). The estimated heritability of renal sinus fat ranged from 43-47% in women and 49-55% in men (Table 2, all p < 0.0001).

**Table 2:** Estimated heritability (h^2^) of renal sinus fat in the Framingham Offspring and Third Generation Cohorts

	Whole Sample*		Women*		Men*	
Renal sinus fat phenotype	h^2^	Standard Error	h^2^	Standard Error	h^2^	Standard Error
Age-adjusted**	0.39	0.04	0.47	0.08	0.51	0.08
Age-BMI adjusted**	0.39	0.04	0.44	0.08	0.49	0.08
Age-VAT adjusted**	0.40	0.04	0.43	0.08	0.55	0.08

Abbreviations: BMI = body mass index; VAT = abdominal visceral adipose tissue

* Whole sample N = 2946; women only sample N = 1489; men only sample N = 1457

** All p-value < 0.0001

### GWAS of renal sinus fat

**Figure 1:**
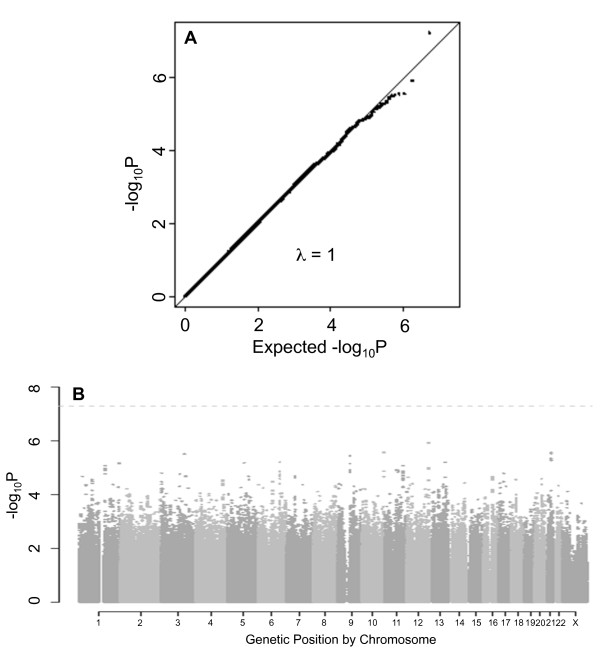
**Renal sinus fat genome-wide association analysis results in the overall sample**. **(a) **Quantile-quantile plot for the genome-wide association analysis of renal sinus fat, adjusted for age, sex, and BMI. **(b) **Manhattan plot of results from the genome-wide association analysis of renal sinus fat, adjusted for age, sex, and BMI. The dotted line represents a -log p-value threshold of 5 × 10^-8^.

Results from the overall GWAS of renal sinus fat are summarized in Figure 1. The quantile-quantile plot of observed versus expected p-values (Figure 1a) suggests that we did not observe p-values below 1 × 10^-8^. As observed in the Manhattan plot (Figure 1b), no SNPs reached genome-wide significance. The 20 top independent SNPs are presented in Table 3, with p-values ranging from 2.6 × 10^-5 ^to 1.2 × 10^-6^.

**Table 3:** Top 20 independent* SNPs associated with renal sinus fat

SNP	Chr	Position (Build 36)	Closest Gene	Effect Allele	Effect Allele Frequency	Imputation Score	P	β (SE)
rs10744391	12	127890891	*GLT1D1*	A	0.29	0.67	1.2E-06	-0.224 (0.046)
rs580140	21	31739378	***TIAM1***	G	0.28	1.01	2.7E-06	-0.184 (0.039)
rs2282335	9	71055752	***TJP2***	A	0.36	0.98	3.6E-06	0.171 (0.037)
rs9375674	6	130258203	*C6orf191*	A	0.46	1.01	6.1E-06	0.158 (0.035)
rs1572050	13	93668766	***GPC6***	A	0.10	0.71	6.3E-06	-0.313 (0.069)
rs17736767	5	98607864	CHD1**	G	0.27	0.74	6.6E-06	-0.208 (0.046)
rs12785341	11	109901068	*ARHGAP20*	C	0.09	0.91	8.3E-06	0.290 (0.065)
rs6686423	1	155704134	*FCRL5*	A	0.31	0.76	8.4E-06	-0.194 (0.044)
rs7311875	12	127859220	***SLC15A4***	G	0.29	1.01	1.0E-05	-0.169 (0.038)
rs907849	11	72001536	***PDE2A***	G	0.23	0.91	1.2E-05	-0.190 (0.043)
rs2511864	11	82196824	*PRCP*	C	0.12	0.97	1.3E-05	0.238 (0.055)
rs332479	3	315440	***CHL1***	C	0.46	1.01	1.6E-05	-0.150 (0.035)
rs3815053	17	32076589	*MRM1*	C	0.48	1.01	1.6E-05	-0.151 (0.035)
rs4529412	7	49243390	VWC2**	T	0.19	0.98	2.1E-05	-0.192 (0.045)
rs1003136	5	127375714	SLC12A2**	A	0.36	0.97	2.1E-05	-0.154 (0.036)
rs6426644	1	20907695	*KIF17*	C	0.36	1.01	2.1E-05	-0.154 (0.036)
rs31093	16	53923677	*IRX6*	G	0.35	1.00	2.2E-05	-0.157 (0.037)
rs9516371	13	93688375	***GPC6***	T	0.33	0.98	2.2E-05	-0.158 (0.037)
rs7325697	13	98915794	*TM9SF2*	T	0.16	0.99	2.3E-05	0.203 (0.048)
rs1543400	20	38896007	MAFB**	C	0.50	0.97	2.6E-05	0.148 (0.035)

β coefficients for the association for sex- and cohort-specific residuals of natural log-transformed renal sinus fat, adjusted for age and BMI.

Nearby genes based on RefSeq genes (build 36). The gene is presented in boldface if the SNP is located within the gene.

* Independent SNPs defined as those with r^2 ^< 0.2 at any locus

** Closest reference gene > 60 Kb away

**Figure 2:**
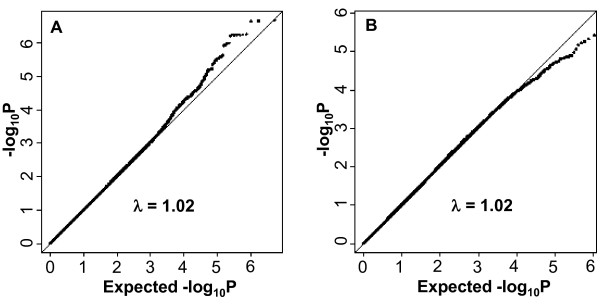
**Renal sinus fat quantile-quantile plots for the genome-wide association analysis by sex**. **(a) **Quantile-quantile plot for the genome-wide association analysis of renal sinus fat in women, adjusted for age and BMI. **(b) **Quantile-quantile plot for the genome-wide association analysis of renal sinus fat in men, adjusted for age and BMI.

**Figure 3:**
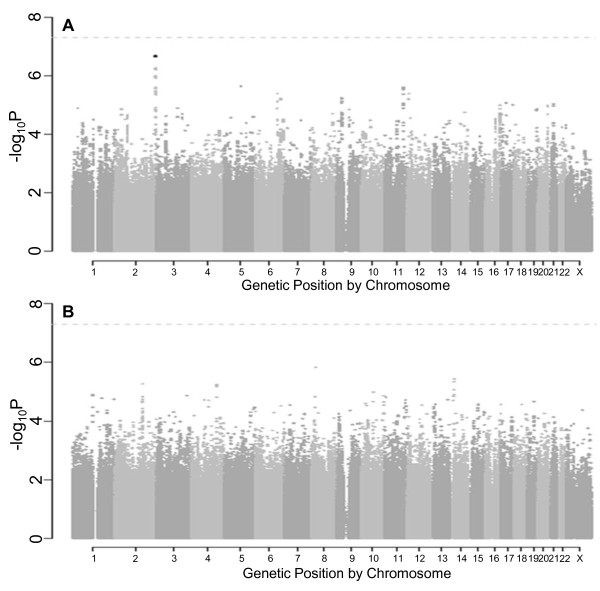
**Renal sinus fat Manhattan plots for the genome-wide association analysis by sex**. **(a) **Manhattan plot of results from the genome-wide association analysis of renal sinus fat in women, adjusted for age and BMI. The dotted line represents a -log p-value threshold of 5 × 10^-8^. **(b) **Manhattan plot of results from the genome-wide association analysis of renal sinus fat in men, adjusted for age and BMI. The dotted line represents a -log p-value threshold of 5 × 10^-8^.

Due to the sex-specific differences observed in regional fat distribution GWAS [24], we performed our GWAS among women and men separately. The quantile-quantile plots in women and men are presented in Figures 2a and 2b, respectively. As observed in the Manhattan plots for women (Figure 3a) and men (Figure 3b), no SNPs reached the genome-wide significant threshold of 5 × 10^-8^. The top five independent SNPs in women and men are presented in Table 4. To further assess potential sex-specific differences in associations, we performed a look-up for the top SNPs from women in the GWAS results in men and the top SNPs from men in the GWAS results in women and observed all p ≥ 0.04 for the opposite sex.

**Table 4:** Top 5 independent* SNPs associated with renal sinus fat in women (above) and men (below)

Women									Results in men for top 5 SNPs observed in women	
**SNP**	**Chr**	**Position** **(Build 36)**	**Closest** **Gene**	**Effect** **Allele**	**Effect** **Allele Frequency**	**Imputation** **Score**	**β**	**P**	**β**	**P**

rs2873333	2	236525509	***CENTG2***	C	0.43	0.69	-0.349	2.1E-07	0.069	0.17
rs17736767	5	98607864	*CHD1*	G	0.27	0.74	-0.340	2.3E-06	-0.080	0.14
rs17111435	11	109950861	*ARHGAP20*	G	0.08	1.00	0.462	2.6E-06	0.083	0.29
rs9375674	6	130258203	*C6orf191*	A	0.46	1.01	0.248	4.1E-06	0.087	0.04
rs2518144	12	8650712	***AICDA***	T	0.45	0.98	0.253	4.1E-06	0.046	0.26

**Men**									Results in women for top 5 SNPs observed in men	

**SNP**	**Chr**	**Position** **(Build 36)**	**Closest** **Gene**	**Effect** **Allele**	**Effect Allele Frequency**	**Imputation** **Score**	**β**	**P**	**β**	**P**

rs2748531	14	23791741	*TGM1*	A	0.13	0.89	-0.299	3.6E-06	-0.126	0.14
rs13414916	2	159662666	***TANC1***	T	0.21	0.99	-0.231	5.4E-06	-0.082	0.23
rs11099681	4	149322904	***NR3C2***	C	0.27	1.01	0.207	5.8E-06	-0.053	0.39
rs12722875	1	116617007	*ATP1A1*	A	0.33	1.01	-0.190	1.3E-05	-0.005	0.93
rs12432394	14	21098781	*OR10G3*	T	0.20	0.78	-0.248	1.4E-05	-0.033	0.67

The β coefficients and p-values each of the top 5 SNPs in the opposite sex are presented in the far right columns.

β coefficients for the association separately in women and men for cohort-specific residuals of natural log-transformed renal sinus fat, adjusted for age and BMI.

Nearby genes based on RefSeq genes (build 36). The gene is presented in boldface if the SNP is located within the gene.

* Independent SNPs defined as those with r^2 ^< 0.2 at any locus

### Candidate gene analysis

Results from the candidate gene analyses of loci associated with renal function, BMI, and WHR are presented in **Tables S1, S2, and S3 **(see Additional File 1). Of the 16 independent renal function SNPs investigated, we observed a nominal, direction-consistent association with renal sinus fat at rs12917707 near *UMOD *(Additional File 1, **Table S1**, p = 0.01). This gene encodes the renal tubular protein uromodulin, and we have previously observed that higher urinary uromodulin concentration is associated with an increased odds of chronic kidney disease as well as with rs4293393, a *UMOD *SNP in linkage disequilibrium with rs12917707 (r^2 ^= 1) [31]. For *UMOD*, the minor T allele of rs12917707 has been previously associated with higher estimated glomerular filtration rate and a lower odds of chronic kidney disease [32]; similarly, we observed lower renal sinus fat for this allele. Of the 32 independent BMI SNPs investigated, we observed a nominal, direction-consistent association with renal sinus fat at rs9816226 near *ETV5 *(Additional File 1, **Table S2**, p = 0.008), and at rs2287019 in *QPCTL *(p = 0.03). We observed two direction-consistent SNPs associated with WHR in *CPEB4 *(Additional File 1, **Table S3**, rs6861681p = 0.02) and in *ZNRF3 *(rs4823006, p = 0.03). However, upon further correction for multiple testing by applying an FDR approach, none of the SNPs in the candidate gene analysis achieved statistical significance (q-values > 0.10).

Finally, we applied the multi-SNP risk score method and observed that when considered in aggregate, the 32 SNPs associated with BMI were nominally associated with renal sinus fat (p = 0.03). In contrast, the set of 16 SNPs associated with renal function (p = 0.55) and the set of 14 SNPs associated with WHR (p = 0.40) were not associated with renal sinus fat.

## Discussion

Renal sinus fat is a heritable trait in the Framingham Heart Study, even after accounting for measures of general and abdominal adiposity. No loci were uncovered in association with renal sinus fat at the genome-wide significant level and none of our individual SNPs derived from recent GWAS analyses for renal function, BMI, and WHR were associated with renal sinus fat after accounting for multiple comparisons. However, our BMI candidate SNP risk score test suggests that the 32 SNPs associated with BMI are, in aggregate, associated with renal sinus fat.

The heritability of renal sinus fat accumulation has not been previously reported. Our estimated heritability of renal sinus fat is consistent with heritability estimates reported for other adiposity measures in the Framingham Heart Study, which include estimates of 37-52% for BMI [33], 41% for waist circumference [19], 36% for abdominal VAT [2], and 57% for abdominal SAT. Our results are notable for their persistence even after accounting for body mass index or abdominal VAT, suggesting that the observed proportion of the variance in renal sinus fat attributable to genotypic variance is not solely attributable to the correlation of renal sinus fat with measures of generalized or abdominal adiposity. Our findings extend the current literature related to the heritability of adiposity traits and provide support for further research into the genetic determinants of renal sinus fat accumulation and, more generally, further exploration of the genetic contributions to ectopic fat accumulation beyond those attributed to overall or abdominal adiposity.

Based on our heritability estimates, we performed a GWAS to potentially identify novel loci associated with renal sinus fat. While we did not observe any loci associated with renal sinus fat at the genome-wide level of significance, we acknowledge that our study was underpowered to detect common genetic variants. The goal of our candidate gene analysis was to identify SNPs that may be jointly associated with renal sinus fat and renal function or other measures of adiposity, which may provide insight into potential mechanisms relating localized renal adiposity with renal function or overall regional fat deposition patterns. However, none of our selected candidate SNPs was individually associated with renal sinus fat after accounting for multiple testing. Of note, when considered in aggregate, a risk score based on the combined 32 BMI SNPs was nominally associated with renal sinus fat, suggesting that our renal sinus fat GWAS results are enriched with SNPs associated with BMI and potentially that the biologic pathways implicated in overall obesity based on these SNPs may also influence ectopic fat accumulation in the renal sinus.

### Strengths and Limitations

A strength of our study is our assessment of renal sinus fat in a well-characterized community-based sample of related individuals, allowing us to estimate the heritability of renal sinus fat while adjusting for the potential effects of general or abdominal adiposity. There are limitations to our study that warrant mention. Our study sample is predominately white, which may limit the generalizability of our results to other ethnic groups. Our measurement of renal sinus fat is based on a single MDCT scan slice within the renal sinus of the right kidney, which may lead to misclassification of renal sinus fat accumulation. We were underpowered to detect modest associations that are characteristic of adiposity-related SNPs [26]. Finally, renal sinus fat is a novel ectopic fat depot that has not been measured in other study samples. Thus, we were unable to potentially combine our sample with other cohorts in order to increase our sample size and improve the power of our analyses.

## Conclusions

Renal sinus fat is a heritable trait, even after accounting for other measures of overall and abdominal adiposity. While our study was underpowered to detect genome-wide significant loci, our heritability estimates provide support for further research into the genetic determinants of renal sinus fat.

## Competing interests

The authors declare that they have no competing interests.

## Authors' contributions

MCF contributed to the conception and design of this study, analysis and interpretation of data, and drafting the manuscript. QY and SJH contributed to the analysis and interpretation of data and revising the article critically for important intellectual content. UH contributed to revising the manuscript critically for important intellectual content. CSF contributed to the conception and design of this study, analysis and interpretation of data, and drafting the manuscript and revising the manuscript for important intellectual content. All authors read and approved the final manuscript.

## Pre-publication history

The pre-publication history for this paper can be accessed here:

http://www.biomedcentral.com/1471-2350/12/148/prepub

## Supplementary Material

Additional file 1 **Additional file **1**includes Supplemental Tables 1, 2, and 3, presenting results from the candidate gene analyses of loci associated with renal function, BMI, and the WHR**. Click here for file
