# Supplementary material for: Heritability and genome-wide association analysis of renal sinus fat accumulation in the Framingham Heart Study
Source: BMC Med Genet. 2011 Nov 1;12:148. doi: 10.1186/1471-2350-12-148 (PMC3243045; doi:10.1186/1471-2350-12-148)
Supplement: Additional file 1 — includes Supplemental Tables 1, 2, and 3, presenting results from the candidate gene analyses of loci associated with renal function, BMI, and the WHR. [file 1471-2350-12-148-S1.DOC]

**Additional file 1: Supplemental tables**

**Supplemental Table 1:** Results from renal function candidate gene analysis*

| **SNP** | **Chr** | **Position (Build 36)** | **Nearby Genes** | **Effect**  **Allele** | **Effect**  **Allele Frequency** | **1-sided**  **P** | **q-value** | **β** | **Standard**  **Error**  **for β** |
| --- | --- | --- | --- | --- | --- | --- | --- | --- | --- |
| rs12917707 | 16 | 20275191 | *PDILT, UMOD* | T | 0.20 | 0.015 | 0.210 | -0.103 | 0.047 |
| rs10109414 | 8 | 23807096 | ***STC1****, NKX3-1* | T | 0.42 | 0.153 | 0.553 | -0.039 | 0.038 |
| rs1260326 | 2 | 27584444 | *HS3ST3B1, CDRT15* | T | 0.46 | 0.173 | 0.553 | 0.035 | 0.037 |
| rs4744712 | 9 | 70624527 | *FAM122A,* ***PIP5K1B*** | A | 0.39 | 0.207 | 0.553 | -0.031 | 0.038 |
| rs13538 | 2 | 73721836 | ***NAT8****, ALMS1* | G | 0.24 | 0.226 | 0.553 | 0.033 | 0.044 |
| rs7805747 | 7 | 151038734 | ***PRKAG2****, RHEB* | A | 0.21 | 0.237 | 0.553 | 0.046 | 0.064 |
| rs17319721 | 4 | 77587871 | ***SHROOM3****, FLJ25770* | A | 0.42 | 0.348 | 0.696 | -0.015 | 0.037 |
| rs653178 | 12 | 110492139 | ***ATXN2****, BRAP* | C | 0.49 | 0.428 | 0.707 | -0.007 | 0.038 |
| rs11959928 | 5 | 39432889 | ***DAB2****, C9* | A | 0.45 | 0.466 | 0.707 | -0.003 | 0.038 |
| rs6420094 | 5 | 176750242 | ***SLC34A1****, PFN3* | G | 0.38 | 0.505 | 0.707 | 0.001 | 0.055 |
| rs1394125 | 15 | 73946038 | ***UBE2Q2****, FBXO22* | A | 0.34 | 0.641 | 0.731 | 0.017 | 0.047 |
| rs267734 | 1 | 149218101 | *ANXA9, LASS2* | C | 0.20 | 0.674 | 0.731 | -0.021 | 0.047 |
| rs881858 | 6 | 43914587 | *VEGFA, MRPS18A* | G | 0.28 | 0.679 | 0.731 | -0.023 | 0.050 |
| rs626277 | 13 | 71245697 | ***DACH1****, LOC440145* | C | 0.44 | 0.800 | 0.774 | 0.036 | 0.043 |
| rs347685 | 3 | 143289827 | *GK5, TFDP2* | C | 0.27 | 0.871 | 0.774 | -0.047 | 0.042 |
| rs12460876 | 19 | 38048731 | ***SLC7A9****, CCDC123* | C | 0.44 | 0.884 | 0.774 | -0.045 | 0.038 |

β coefficients are for the association for residuals of natural log-transformed renal sinus fat, adjusted for age and sex. Nearby genes based on RefSeq genes (build 36). The gene is presented in boldface if the SNP is located within the gene.

*Candidate gene SNPs selected from Kottgen et al. Nat Genet. 2010 May;42(5):376-84.

**Supplemental Table 2: Results from body mass index candidate gene analysis***

| **SNP** | **Chr** | **Position (Build 36)** | **Closest Genes** | **Effect**  **Allele** | **Effect**  **Allele Frequency** | **1-sided P** | **q-value** | **β** | **Standard Error for β** |
| --- | --- | --- | --- | --- | --- | --- | --- | --- | --- |
| rs9816226 | 3 | 187317193 | *ETV5, DGKG* | A | 0.192 | 0.008 | 0.144 | -0.115 | 0.048 |
| rs2287019 | 19 | 50894012 | ***QPCTL****, GIPR* | T | 0.174 | 0.032 | 0.245 | -0.109 | 0.059 |
| rs1558902 | 16 | 52361075 | ***FTO****, RPGRIP1L* | A | 0.423 | 0.060 | 0.245 | 0.060 | 0.038 |
| rs11847697 | 14 | 29584863 | *PRKD1, KIAA1333* | T | 0.045 | 0.079 | 0.245 | 0.137 | 0.097 |
| rs7359397 | 16 | 28793160 | *SH2B1, ATP2A1* | T | 0.360 | 0.087 | 0.245 | 0.054 | 0.040 |
| rs2241423 | 15 | 65873892 | ***MAP2K5*** *LBXCOR1* | A | 0.214 | 0.090 | 0.245 | -0.060 | 0.045 |
| rs7138803 | 12 | 48533735 | *BCDIN3D, FAIM2* | A | 0.384 | 0.101 | 0.245 | 0.051 | 0.040 |
| rs10150332 | 14 | 79006717 | ***NRXN3****, DIO2* | C | 0.200 | 0.109 | 0.245 | 0.056 | 0.046 |
| rs12444979 | 16 | 19841101 | *GPRC5B, IQCK* | T | 0.149 | 0.209 | 0.348 | -0.042 | 0.052 |
| rs887912 | 2 | 59156381 | *FANCL, VRK2* | T | 0.303 | 0.211 | 0.348 | 0.033 | 0.041 |
| rs2112347 | 5 | 75050998 | *C5orf37, POLK* | G | 0.371 | 0.216 | 0.348 | -0.030 | 0.038 |
| rs2815752 | 1 | 72585028 | *NEGR1, ZRANB2* | G | 0.377 | 0.263 | 0.348 | -0.024 | 0.038 |
| rs4836133 | 5 | 124360002 | *ZNF608, CSNK1G3* | A | 0.509 | 0.267 | 0.348 | 0.024 | 0.038 |
| rs571312 | 18 | 55990749 | *MC4R, PMAIP1* | A | 0.207 | 0.288 | 0.348 | 0.026 | 0.046 |
| rs3810291 | 19 | 52260843 | ***C19orf7****, TMEM160* | G | 0.344 | 0.303 | 0.348 | -0.023 | 0.045 |
| rs4929949 | 11 | 8561169 | ***STK33****, RPL27A* | T | 0.492 | 0.309 | 0.348 | -0.019 | 0.038 |
| rs713586 | 2 | 25011512 | *RBJ, ADCY3* | T | 0.529 | 0.355 | 0.354 | -0.014 | 0.038 |
| rs2890652 | 2 | 142676401 | *LRP1B, KYNU* | C | 0.140 | 0.358 | 0.354 | 0.020 | 0.054 |
| rs987237 | 6 | 50911009 | ***TFAP2B****, TFAP2D* | G | 0.168 | 0.385 | 0.354 | 0.014 | 0.049 |
| rs10968576 | 9 | 28404339 | ***LINGO2****, C9orf72* | G | 0.301 | 0.410 | 0.354 | 0.009 | 0.040 |
| rs13107325 | 4 | 103407732 | ***SLC39A8****, BANK1* | T | 0.081 | 0.413 | 0.354 | 0.015 | 0.068 |
| rs3817334 | 11 | 47607569 | ***MTCH2****, AGBL2* | T | 0.420 | 0.445 | 0.354 | 0.005 | 0.037 |
| rs206936 | 6 | 34410847 | ***NUDT3****, RPS10* | G | 0.208 | 0.452 | 0.354 | 0.006 | 0.046 |
| rs2867125 | 2 | 612827 | *TMEM18, LOC391343* | T | 0.174 | 0.537 | 0.403 | 0.005 | 0.049 |
| rs543874 | 1 | 176156103 | *SEC16B, RASAL2* | G | 0.180 | 0.581 | 0.405 | -0.010 | 0.048 |
| rs1555543 | 1 | 96717385 | *PTBP2, DPYD* | A | 0.426 | 0.585 | 0.405 | 0.008 | 0.037 |
| rs1514175 | 1 | 74764232 | ***TNNI3K****, C1orf173* | A | 0.420 | 0.706 | 0.471 | -0.021 | 0.038 |
| rs10938397 | 4 | 44877284 | *GNPDA2, GUF1* | G | 0.422 | 0.781 | 0.502 | -0.033 | 0.042 |
| rs29941 | 19 | 39001372 | *KCTD15, CHST8* | A | 0.326 | 0.818 | 0.508 | 0.036 | 0.039 |
| rs13078807 | 3 | 85966840 | ***CADM2****, VGLL3* | G | 0.214 | 0.882 | 0.529 | -0.056 | 0.047 |
| rs10767664 | 11 | 27682562 | ***BDNF****, LIN7C* | T | 0.212 | 0.977 | 0.552 | 0.091 | 0.046 |
| rs4771122 | 13 | 26918180 | ***MTIF3****, LNX2* | G | 0.231 | 0.982 | 0.552 | -0.096 | 0.046 |

β coefficients are for the association for residuals of natural log-transformed renal sinus fat, adjusted for age and sex. Nearby genes based on RefSeq genes (build 36). The gene is presented in boldface if the SNP is located within the gene.

*Candidate gene SNPs selected from Speliotes et al. Nat Genet. 2010 Nov;42(11):937-48.

Supplemental Table 3: Results from waist-to-hip ratio adjusted for body mass index candidate gene analysis*

| **SNP** | **Chr** | **Position (Build 36)** | **Closest Genes** | **Effect**  **Allele** | **Effect**  **Allele Frequency** | **1-sided P** | **q-value** | **β** | **Standard Error**  **for β** |
| --- | --- | --- | --- | --- | --- | --- | --- | --- | --- |
| rs6861681 | 5 | 173295064 | ***CPEB4****, HMP19* | A | 0.30 | 0.022 | 0.108 | 0.086 | 0.043 |
| rs4823006 | 22 | 27781671 | ***ZNRF3****, C22orf31* | G | 0.46 | 0.027 | 0.108 | -0.072 | 0.038 |
| rs984222 | 1 | 119305366 | ***TBX15****, WARS2* | C | 0.41 | 0.106 | 0.262 | -0.048 | 0.038 |
| rs718314 | 12 | 26344550 | *ITPR2, SSPN* | G | 0.25 | 0.131 | 0.262 | 0.049 | 0.044 |
| rs1443512 | 12 | 52628951 | *HOXC13, HOXC12* | A | 0.23 | 0.210 | 0.336 | 0.035 | 0.044 |
| rs1294421 | 6 | 6688148 | *LY86, RREB1* | T | 0.39 | 0.276 | 0.336 | -0.023 | 0.038 |
| rs1055144 | 7 | 25837634 | *NFE2L3, HNRNPA2B1* | T | 0.19 | 0.326 | 0.336 | 0.021 | 0.047 |
| rs1011731 | 1 | 170613171 | ***DNM3****, C1orf105* | G | 0.44 | 0.356 | 0.336 | 0.014 | 0.037 |
| rs9491696 | 6 | 127494332 | ***RSPO3****, RNF146* | C | 0.53 | 0.378 | 0.336 | -0.011 | 0.037 |
| rs4846567 | 1 | 217817340 | *SLC30A10, LYPLAL1* | T | 0.29 | 0.480 | 0.384 | -0.002 | 0.041 |
| rs6784615 | 3 | 52481466 | ***NISCH****, TNNC1* | C | 0.06 | 0.792 | 0.536 | 0.064 | 0.079 |
| rs10195252 | 2 | 165221337 | *COBLL1, GRB14* | C | 0.39 | 0.819 | 0.536 | 0.035 | 0.038 |
| rs6795735 | 3 | 64680405 | *ADAMTS9, PRICKLE2* | T | 0.46 | 0.925 | 0.536 | 0.053 | 0.037 |
| rs6905288 | 6 | 43866851 | *VEGFA, MRPS18A* | G | 0.42 | 0.938 | 0.536 | 0.059 | 0.038 |

β coefficients are for the association for residuals of natural log-transformed renal sinus fat, adjusted for age and sex. Nearby genes based on RefSeq genes (build 36). The gene is presented in boldface if the SNP is located within the gene.

*Candidate gene SNPs selected from Heid et al. Nat Genet. 2010 Nov;42(11):949-60.
